# A case report of ethanol infusion in the vein of Marshall using the right jugular vein approach

**DOI:** 10.1093/ehjcr/ytaa260

**Published:** 2020-08-26

**Authors:** Severine Philibert, Denis Amet, Margaux De Chirac, Gabriel Laurent

**Affiliations:** HEGP, 20 rue Leblanc, 75015, Paris, France; HEGP, 20 rue Leblanc, 75015, Paris, France; Biosense Webster Inc, Johnson & Johnson, Irvine, CA, USA; CHU Dijon, 14 rue Gaffarel, 21000, Dijon

**Keywords:** Vein of Marshall (VOM), Jugular vein approach, Ablation, Case report

## Abstract

**Background:**

Ethanol infusion has recently been described as a curative strategy for certain peri-mitral flutters by blocking electrical conduction across the mitral isthmus along with the Marshall bundle. The present case showed that a right jugular vein approach, less described, may be a good choice when performing an ethanol infusion in the vein of Marshall (VOM).

**Case summary:**

A 45-year-old man was admitted to our unit for dyspnoea associated with an atypical atrial flutter with a cycle length of 320 ms. The left atrial activation map showed a peri-mitral counter-clockwise circuit. The atrial flutter cycle length went up to 345 ms once an endocardial and epicardial point-by point-ablation of the mitral line was completed. At this stage, a new activation map showed that the mitral line was still permeable with an epicardial conduction bridge through the VOM. We decided to use an ethanol infusion for the ablation of the VOM. The coronary sinus could not be thoroughly catheterized due to a winding and angular shape so we decided to try a right jugular vein approach. A total of 9 mL of ethanol was injected into the VOM. A final venogram showed the diffusion of ethanol around the VOM. Sinus rhythm was restored during the last ethanol infusion. A new voltage map confirmed the completion of the mitral line, and we confirmed the bidirectional block.

**Discussion:**

The present case showed that a right jugular vein approach may be a good choice when catheterizing and performing an ethanol infusion in the VOM.

**Learning points**

- Ethanol infusion inside the vein of Marshall may be a complementary technique to complete mitral isthmus conduction block after endocardial and epicardial radiofrequency ablation.
- Right jugular vein approach may be of interest when the femoral approach is not feasible.

## Introduction

The ligament or vein of Marshall (VOM) is anatomical and electrical structures that are sometimes involved in macro-reentry and micro-reentry circuits. By bridging endocardial activation at the mitral isthmus, the VOM may be responsible for peri-mitral flutters that occur after atrial fibrillation (AF) ablation.^1^^,^^2^ Ethanol (EtOH) infusion has recently been described as a curative strategy for certain peri-mitral flutters by blocking electrical conduction across the mitral isthmus along with the Marshall bundle.^3^ The standard way of cannulating the VOM is through a femoral approach, which has been detailed in several case reports.^4^^,^^5^ The jugular approach is also possible, but it has been less described.^5^^,^^6^

## Timeline

**Table:** 

Seven months before	Transthoracic echocardiography revealed predominantly septal and posterior hypertrophic cardiopathy with intraventricular obstruction of flow (basic gradient at 45 mmHg up to 60 mmHg after Valsalva manoeuvres). The left atrium was dilated (surface = 53 mL/m^2^)
	Patient underwent atrial fibrillation ablation (isolation of pulmonary veins and roof line)
Initial presentation	Symptomatic atypical atrial flutter
Hospitalization	Invasive electrophysiological study and ablation procedure with diagnosis of peri-mitral flutter. No conduction block with endocardial and epicardial application, ethanol infusion of the vein of Marshall with the achievement of bidirectional block.
1-month post-ablation	Patient asymptomatic without recurrence of arrhythmia.

## Case presentation 

**Figure 1:**
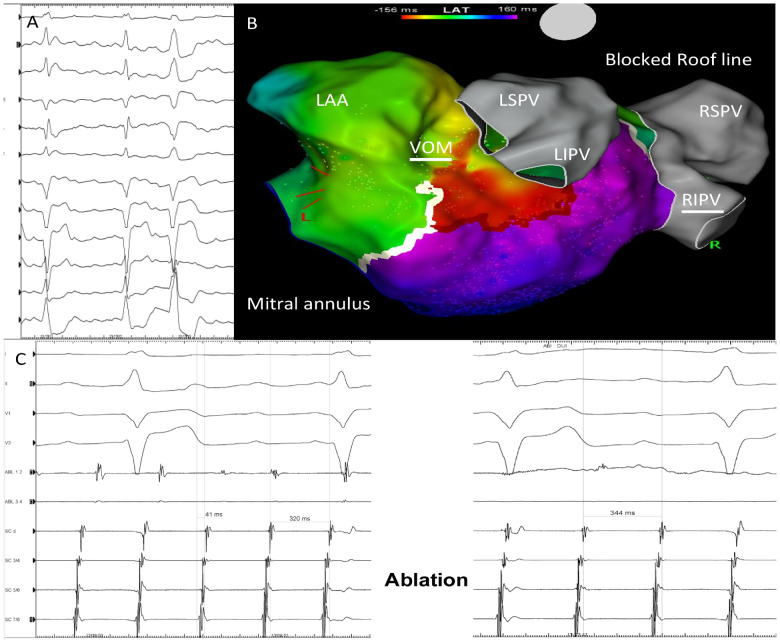
(*A*) 12 lead electrocardiogram. Atypical atrial flutter with positive p waves at inferior and precordial leads. (*B*) Activation map during flutter using Carto system. Modified postero-anterior (PA) view. Incomplete conduction block through the posterior part of the mitral isthmus due to the vein of Marshall [the anterior part which close to the mitral annulus is blocked: the white line corresponds to the ‘Extended Early Meets Late’ algorithm which was set up at 75–25% of the cycle length (CL)]. (*C*) Initial CL was 320 ms (left), with activation time of 41 ms, and went up to 345 ms (right) after endocardial and epicardial point-by point-ablation of the mitral line, without any changes in the activation sequence and activation time.

A 45-year-old man was admitted to our unit for dyspnoea associated with an atypical atrial flutter with positive p waves in the inferior and precordial leads. He was already being treated with Disopyramide LP 125 mg and Bisoprolol 2.5 mg per day (*Figure 1*). He had undergone an AF ablation procedure 7 months previously, during which a micro-reentry was ablated on the roof of the left atrium (LA) near the right superior pulmonary vein (PV). The procedure was then completed with antral PV isolation, and a complete roofline block was done in order to restore sinus rhythm.

Transthoracic echocardiography revealed predominantly septal and posterior hypertrophic cardiopathy with intraventricular obstruction of flow (basic gradient at 45 mmHg up to 60 mmHg after Valsalva manoeuvres). The LA was found to be dilated, with a measured surface of 53 mL/m^2^. Computed tomography scan imaging showed a moderately dilated LA (42 cm^2^) with normal PV anatomy and no thrombus in the left atrial appendage (LAA).

After informed consent was obtained, the patient underwent a new endocardial electrophysiology procedure. We used the same 3D navigation system as for the first procedure (CARTO 3D, Biosense Webster Inc., Johnson & Johnson, Irvine, CA, USA). At the beginning of the procedure, the rhythm was a stable atrial flutter with a cycle length of 320 ms. We observed a proximal to distal activation on the coronary sinus (CS) with activation time at 41 ms. A Pentaray catheter inserted through a transseptal puncture helped to identify low voltage areas (scar tissue < 0.2 mV) on the roof and in the right superior pulmonary vein (RSPV). The three other veins were connected and active. The LA activation map showed a peri-mitral counter-clockwise circuit, while the roofline was blocked. A radiofrequency (RF) ablation catheter, Thermocool Smart Touch SF (Biosense Webster, Diamond Bar, CA, USA), was used endocardially with a set-up power at 40 W and a saline irrigation rate of 17 mL/min and an ablation index goal of 450 at the anterior wall and roof and 370 at the posterior wall. We first re-isolated the three active PVs which did not change the cycle length. The atrial flutter cycle length went up to 345 ms once an endocardial and epicardial point-by-point ablation of the mitral line was completed.

**Figure 2:**
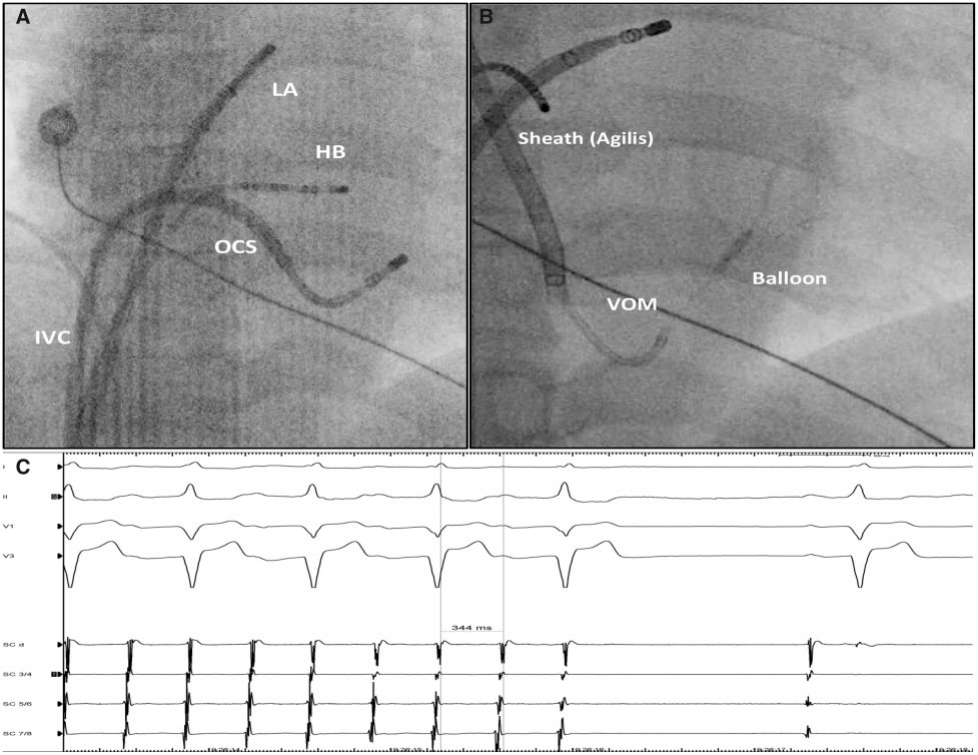
(*A*) Fluoroscopic image PA view, showing the partial catheterization of the coronary sinus from the femoral approach. (*B*) Fluoroscopic image left anterior oblique (LAO) 30° view. Venogram of the VOM using an inflated 1.5 mm balloon placed inside the vein. The iodine contrast is trapped inside the vein of Marshall (injection shows dye ‘hang up’). Black arrows indicate extravasation of contrast. (*C*) Electrogram recordings obtained during the last ethanol infusion inside the vein of Marshall. Sinus rhythm was immediately restored. HB, His bundle; IVC, inferior vena cava; LA, left atrium; OCS, ostium of CS; SCd, distal coronary sinus; SC 7-8, proximal CS; VOM, vein of Marshall.

**Figure 3:**
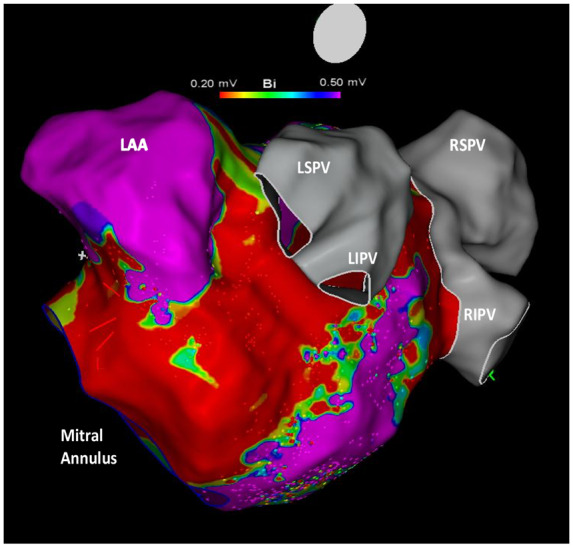
(*A*) Voltage map performed after endocardial and epicardial mitral line ablation and before ethanol infusion in the vein of Marshall. A non-complete low voltage area is visible along the mitral line. (*B*) Voltage map after ethanol infusion in the vein of Marshall. A large low voltage band can be seen along the mitral line. LAA, left atrial appendage; LIPV, left inferior pulmonary vein; LSPV, left superior pulmonary vein; RIPV, right inferior pulmonary vein; RSPV, right superior pulmonary vein.

**Figure 4:**
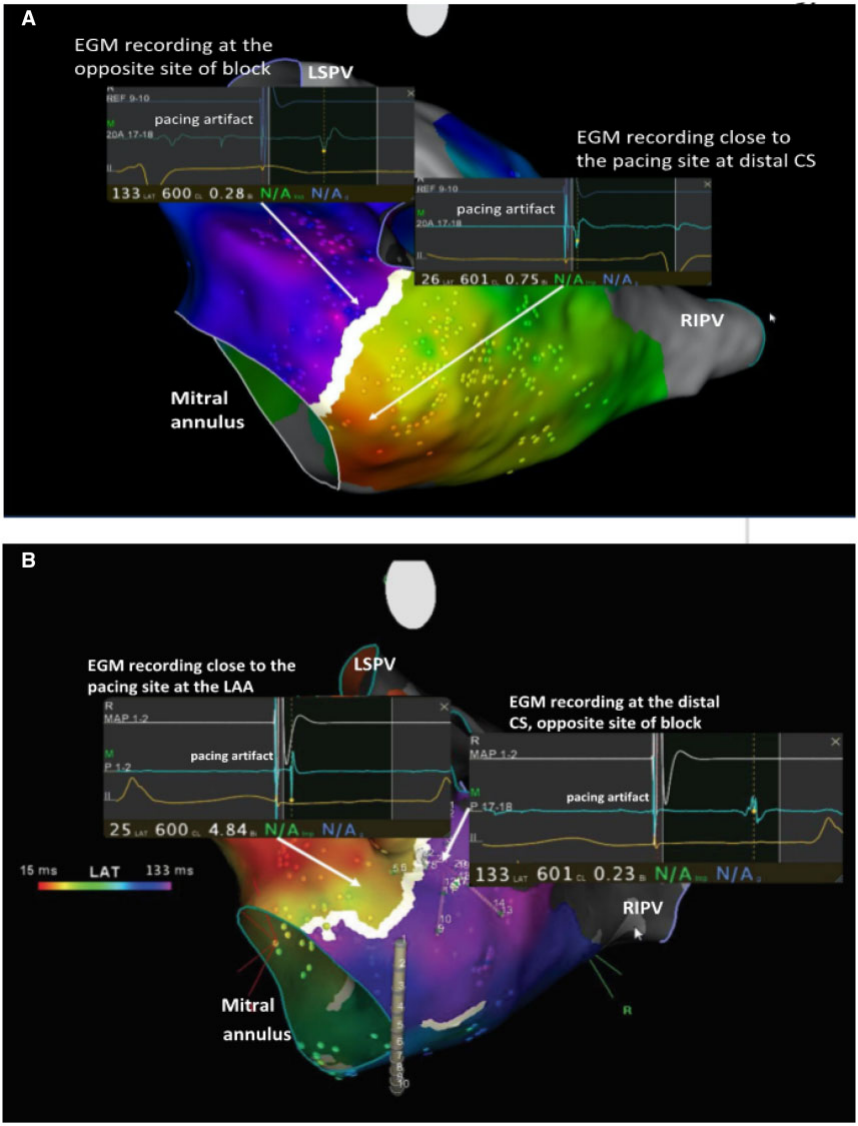
(*A*) Counter-clockwise peri-mitral block evidenced by pacing at the distal part of the coronary sinus while recording at the opposite site. (*B*) Clockwise peri-mitral block evidence by pacing at the left atrial appendage while recording at the distal coronary sinus.

At this stage, a new activation map showed that the mitral line was still permeable with an epicardial conduction bridge through the VOM (*Figure 1*). We then decided to use an ethanol infusion for the ablation of the VOM. The coronary sinus could not be thoroughly catheterized with femoral approach due to a winding and angular shape (*Figure 2A*) neither with a deflectable decapolar catheter Xtrem (Microport, Shangai, China) nor with two different sheaths (SL0 Abbott, Saint Paul, Minnesota, USA and Agilis™ NxT Steerable Introducer, Abbott, Saint Paul, MN, USA). So, we decided to try a right jugular vein approach with echo-guided puncture using an Agilis™ NxT Steerable Introducer (Abbott, Saint Paul, MN, USA). We had no problem finding the VOM ostium using a 5 Fr angiography catheter (5 Fr left internal mammary artery; Medtronic, Minneapolis, MN, USA) that was inserted into the CS via the steerable sheath. We then performed a selective venogram of the vein using a 1.5 mm balloon (1.5–2.5 mm diameter and 6–15 mm length, Abbott) that was progressively inflated (from 2 to 6 atm) inside the vein over an angioplasty wire (Whisper 0.014, Abbott). After we confirmed the complete occlusion of the VOM by injecting 1 mL of contrast medium, 3 mL of ethanol (96% ethanol 10 mL) was slowly injected over a period of 1 minute, and venography of the VOM was repeated. Following the initial injection and using the same technique, a complementary injection was performed with 3 mL of ethanol inside another branch of the VOM. A total of 9 mL of ethanol was used as a maximum dose. A final venogram showed the diffusion of ethanol around the VOM (*Figure 2B*). Sinus rhythm was restored during the last ethanol infusion (*Figure 2C*). A new voltage map confirmed the completion of the mitral line (*Figure 3*). Activation maps sequentially paced in the distal CS and in the LAA confirmed a bidirectional atrial block (*Figure 4*).

At 1-month post-ablation, the patient was asymptomatic without recurrence of arrhythmia.

## Discussion

Ethanol infusion is a new alternative approach to treat peri-mitral atrial flutters which are refractory to mitral isthmus endocardial RF ablation.^6^ The ligament of Marshall is a unique anatomical structure electrically insulated by surrounding adipose tissue or vessels protecting it from endocardial RF energy applications.^7^ Sometimes, it is still a vein (VOM) that can be followed retrogradely from its insertion into the main body of the CS, at the epicardial aspect of the mitral annulus, to the top of the ridge between the LA appendage and the left-sided PVs. Although this vein is usually too small to be targeted by a conventional ablation catheter, the atrial tissue drained by it may be efficiently ablated by retrograde EtOH infusion via the CS. Whereas the femoral vein approach is the regular technique to cannulate the VOM with an EtOH infusion success rate ranging from 89% to 92%,^5^^,^^8^ to date, there are only a few reports using the jugular vein approach as a surrogate. Some authors are using the jugular vein approach as a regular technique which seems to be as efficient as the femoral vein approach (86%).^9^ In our case, we have shown that this technique may be of interest whenever the femoral approach is not suitable. In addition, the right jugular vein approach may have several advantages: (i) the steerable sheath can also be useful to insert an RF ablation catheter in order to complete the epicardial conduction block after ethanol infusion if needed, (ii) one operator can perform the EtOH infusion while the other one is confirming the presence of a bidirectional endocardial conduction block, and (iii) the echo-guided right jugular vein puncture may lead to simple post-operative follow-up.

## Conclusion

The present case showed that a right jugular vein approach may be a good choice when catheterizing and performing an ethanol infusion in the VOM. This recently described technique appears to be suitable for obtaining a conduction block through the mitral isthmus. However, it must be noted that endocardial RF applications were also needed to complete the block.

## Lead author biography 

**Figure:**
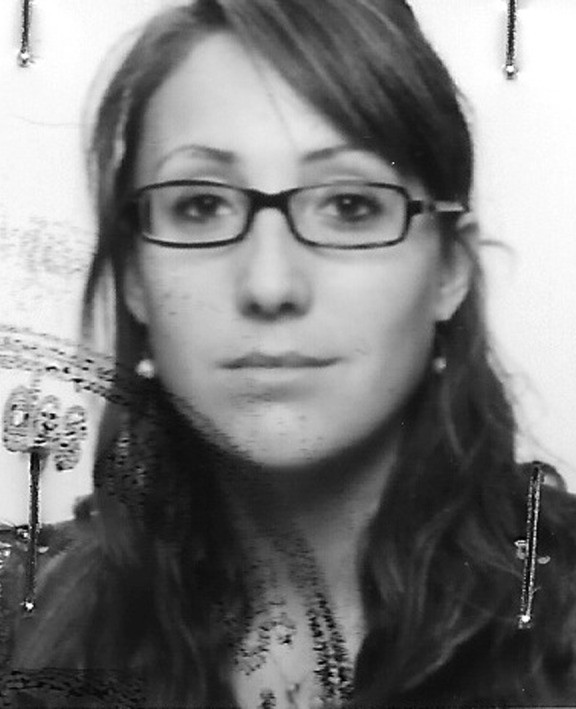


Dr Philibert Severine MD is a cardiologist specialized in rhythmology working at the Europeen Georges Pompidou Hospital in Paris, France. Born in France, I received local state education in cardiology at the University of Burgundy (France) during my internship. My two-year post-doctoral fellowship took place at the South Cardiac institute of Massy (France) and the second year at the Heart institute of Montreal, Quebec (Canada). After Montreal, I came back to Paris where I began to practice invasive complex electrophysiology and cardiac stimulation.

## Supplementary material

Supplementary material is available at *European Heart Journal - Case Reports* online.

**Slide sets:** A fully edited slide set detailing this case and suitable for local presentation is available online as Supplementary data. 

**Consent:** The author/s confirm that written consent for submission and publication of this case report including image(s) and associated text has been obtained from the patient in line with COPE guidelines.

**Conflict of interest:** none declared. 

## Supplementary Material

ytaa260_Supplementary_Data
