# Supplementary material for: A case report of ethanol infusion in the vein of Marshall using the right jugular vein approach
Source: Eur Heart J Case Rep. 2020 Aug 26;4(5):1–6. doi: 10.1093/ehjcr/ytaa260 (PMC7649458; doi:10.1093/ehjcr/ytaa260)
Supplement: ytaa260_Supplementary_Data [file ytaa260_supplementary_data.zip › ytaa260_Supplementary_Data/ytaa260_Slide_Set.pptx]

## Slide 1
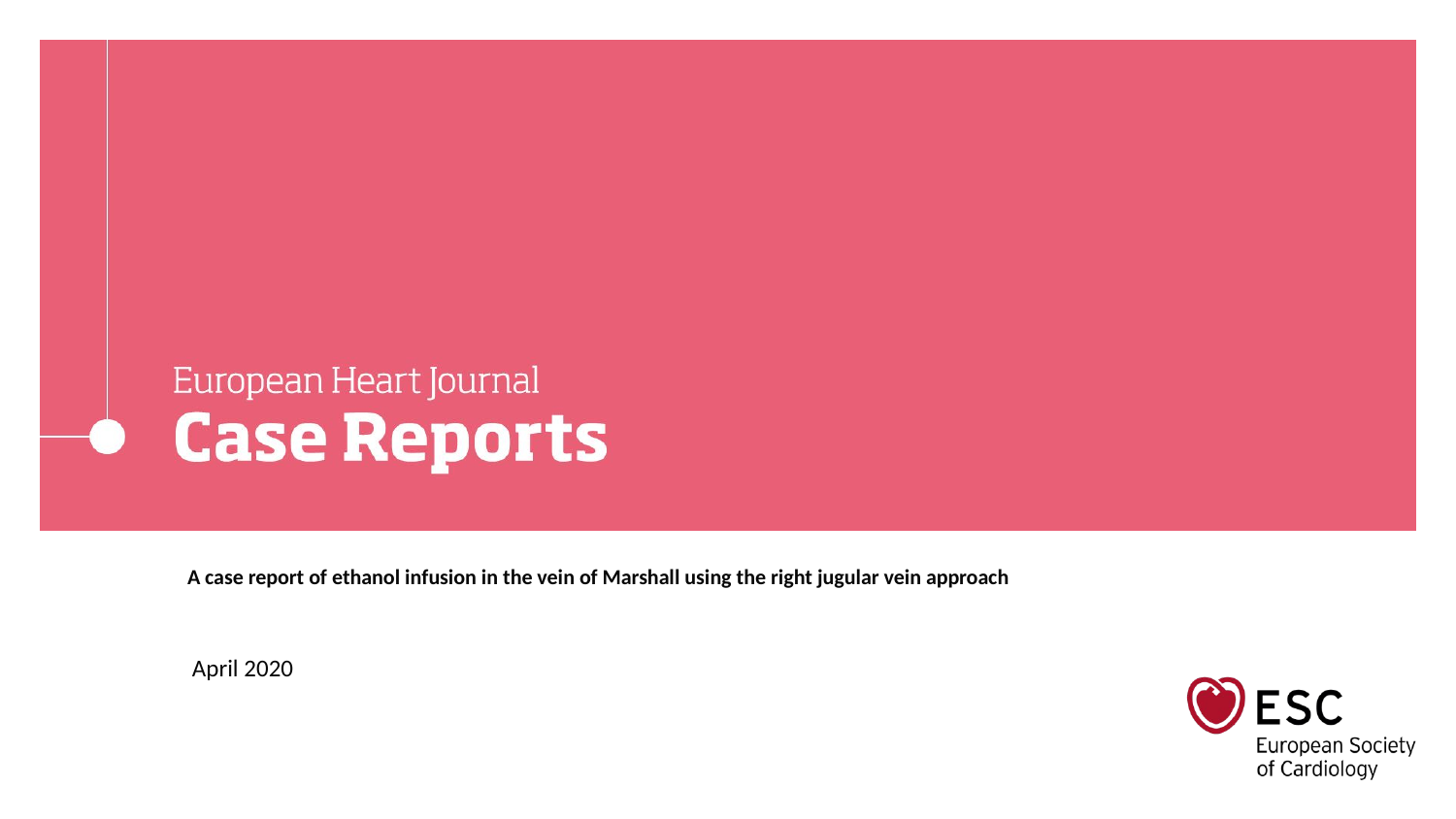

# A case report of ethanol infusion in the vein of Marshall using the right jugular vein approach
April 2020

## Slide 2
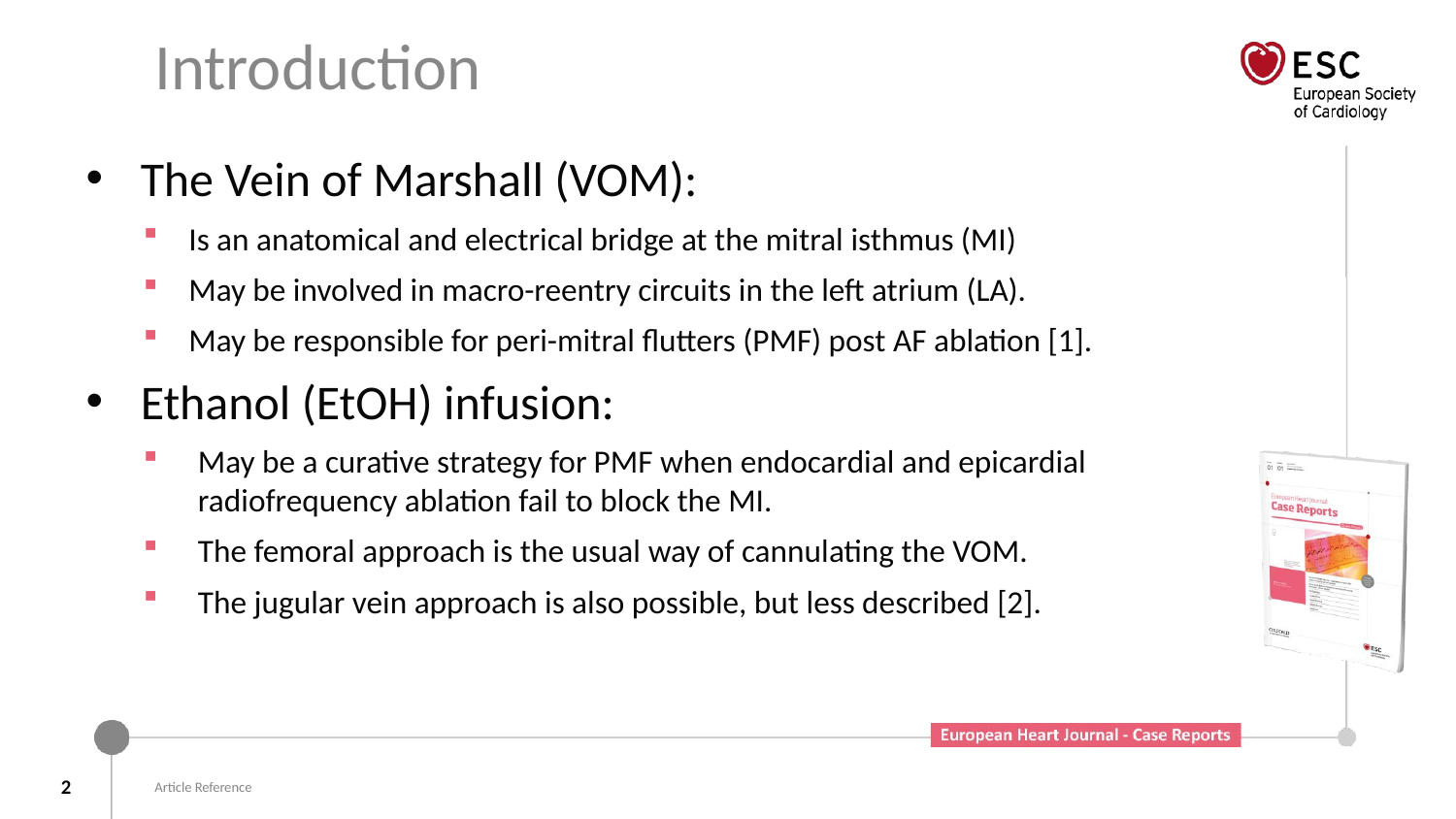

# Introduction
The Vein of Marshall (VOM):
Is an anatomical and electrical bridge at the mitral isthmus (MI)
May be involved in macro-reentry circuits in the left atrium (LA).
May be responsible for peri-mitral flutters (PMF) post AF ablation [1].
Ethanol (EtOH) infusion:
May be a curative strategy for PMF when endocardial and epicardial radiofrequency ablation fail to block the MI.
The femoral approach is the usual way of cannulating the VOM.
The jugular vein approach is also possible, but less described [2].
2
Article Reference

## Slide 3
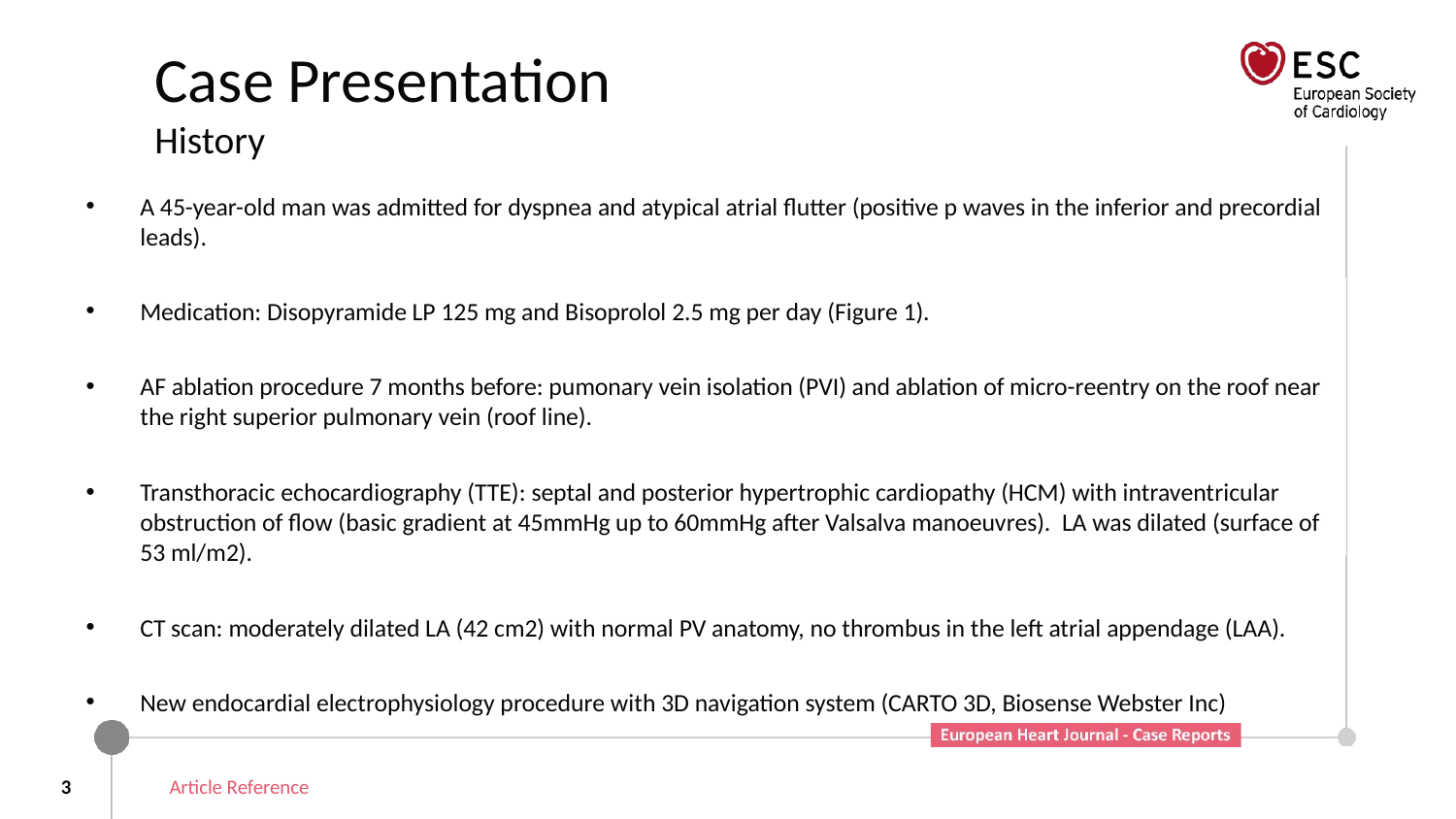

# Case Presentation History
A 45-year-old man was admitted for dyspnea and atypical atrial flutter (positive p waves in the inferior and precordial leads).
Medication: Disopyramide LP 125 mg and Bisoprolol 2.5 mg per day (Figure 1).
AF ablation procedure 7 months before: pumonary vein isolation (PVI) and ablation of micro-reentry on the roof near the right superior pulmonary vein (roof line).
Transthoracic echocardiography (TTE): septal and posterior hypertrophic cardiopathy (HCM) with intraventricular obstruction of flow (basic gradient at 45mmHg up to 60mmHg after Valsalva manoeuvres). LA was dilated (surface of 53 ml/m2).
CT scan: moderately dilated LA (42 cm2) with normal PV anatomy, no thrombus in the left atrial appendage (LAA).
New endocardial electrophysiology procedure with 3D navigation system (CARTO 3D, Biosense Webster Inc)
3
Article Reference

## Slide 4
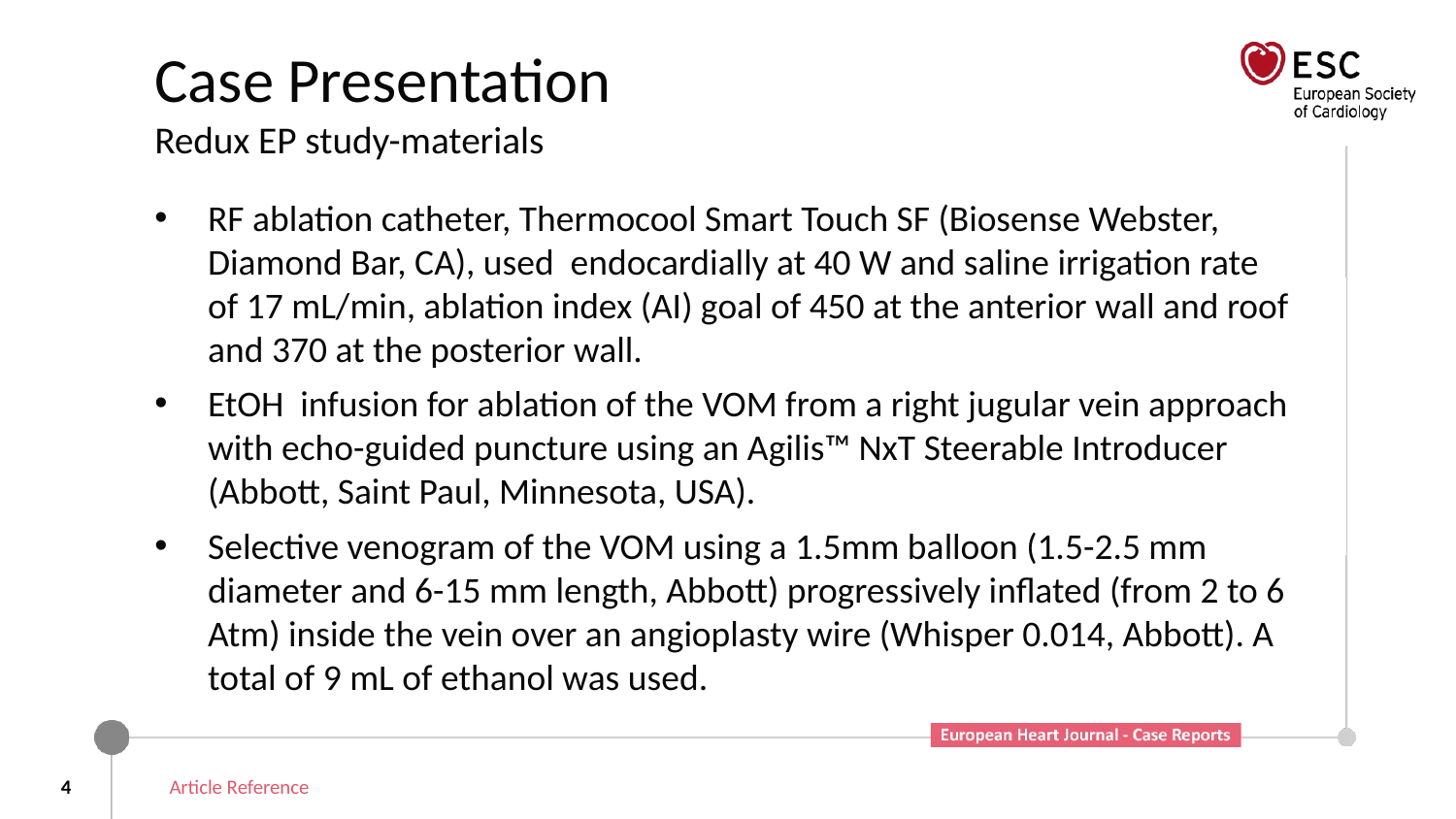

# Case Presentation Redux EP study-materials
RF ablation catheter, Thermocool Smart Touch SF (Biosense Webster, Diamond Bar, CA), used endocardially at 40 W and saline irrigation rate of 17 mL/min, ablation index (AI) goal of 450 at the anterior wall and roof and 370 at the posterior wall.
EtOH infusion for ablation of the VOM from a right jugular vein approach with echo-guided puncture using an Agilis™ NxT Steerable Introducer (Abbott, Saint Paul, Minnesota, USA).
Selective venogram of the VOM using a 1.5mm balloon (1.5-2.5 mm diameter and 6-15 mm length, Abbott) progressively inflated (from 2 to 6 Atm) inside the vein over an angioplasty wire (Whisper 0.014, Abbott). A total of 9 mL of ethanol was used.
4
Article Reference

## Slide 5
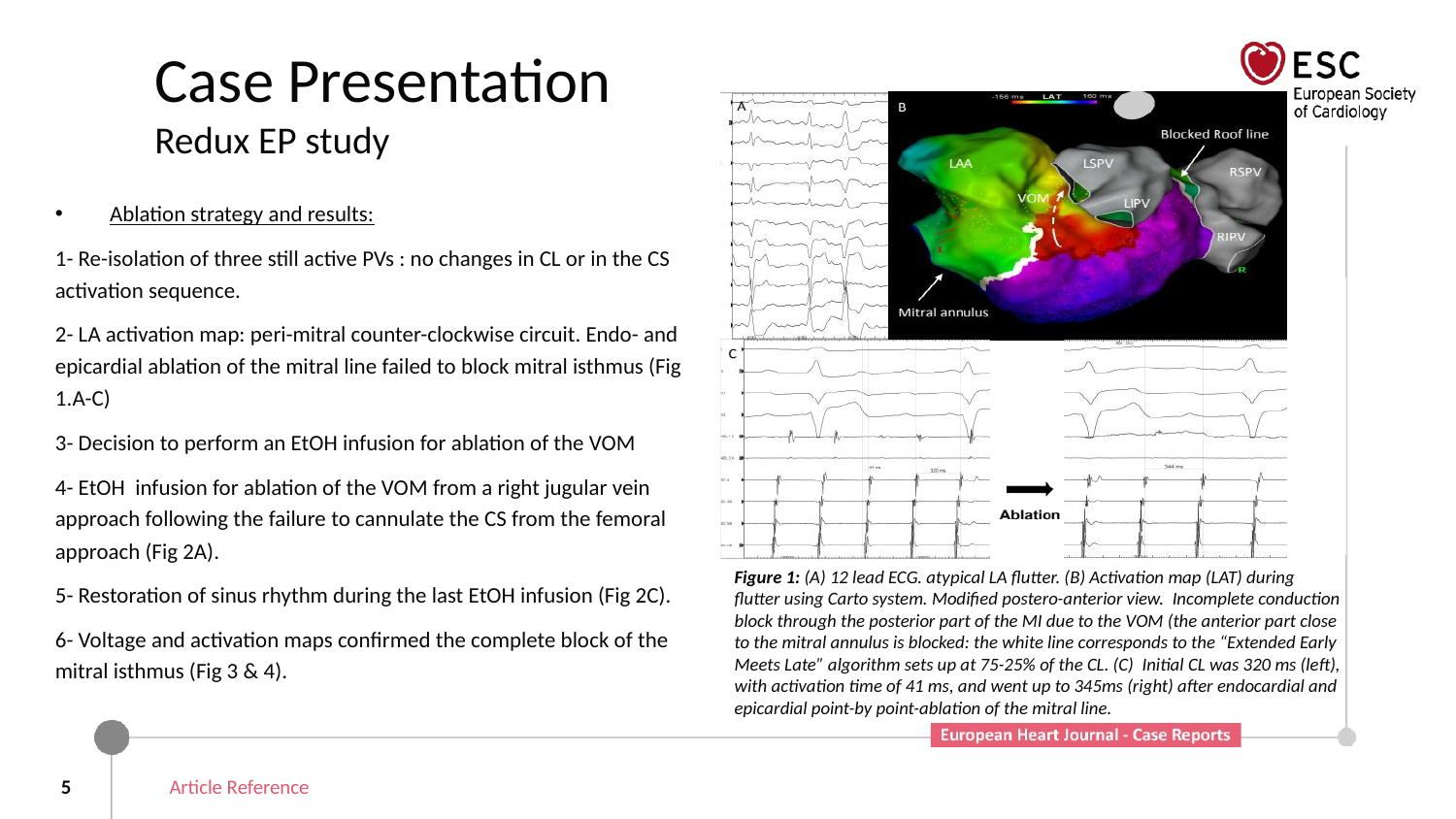

# Case Presentation Redux EP study
Ablation strategy and results:
1- Re-isolation of three still active PVs : no changes in CL or in the CS activation sequence.
2- LA activation map: peri-mitral counter-clockwise circuit. Endo- and epicardial ablation of the mitral line failed to block mitral isthmus (Fig 1.A-C)
3- Decision to perform an EtOH infusion for ablation of the VOM
4- EtOH infusion for ablation of the VOM from a right jugular vein approach following the failure to cannulate the CS from the femoral approach (Fig 2A).
5- Restoration of sinus rhythm during the last EtOH infusion (Fig 2C).
6- Voltage and activation maps confirmed the complete block of the mitral isthmus (Fig 3 & 4).
Figure 1: (A) 12 lead ECG. atypical LA flutter. (B) Activation map (LAT) during flutter using Carto system. Modified postero-anterior view. Incomplete conduction block through the posterior part of the MI due to the VOM (the anterior part close to the mitral annulus is blocked: the white line corresponds to the “Extended Early Meets Late” algorithm sets up at 75-25% of the CL. (C) Initial CL was 320 ms (left), with activation time of 41 ms, and went up to 345ms (right) after endocardial and epicardial point-by point-ablation of the mitral line.
5
Article Reference

## Slide 6
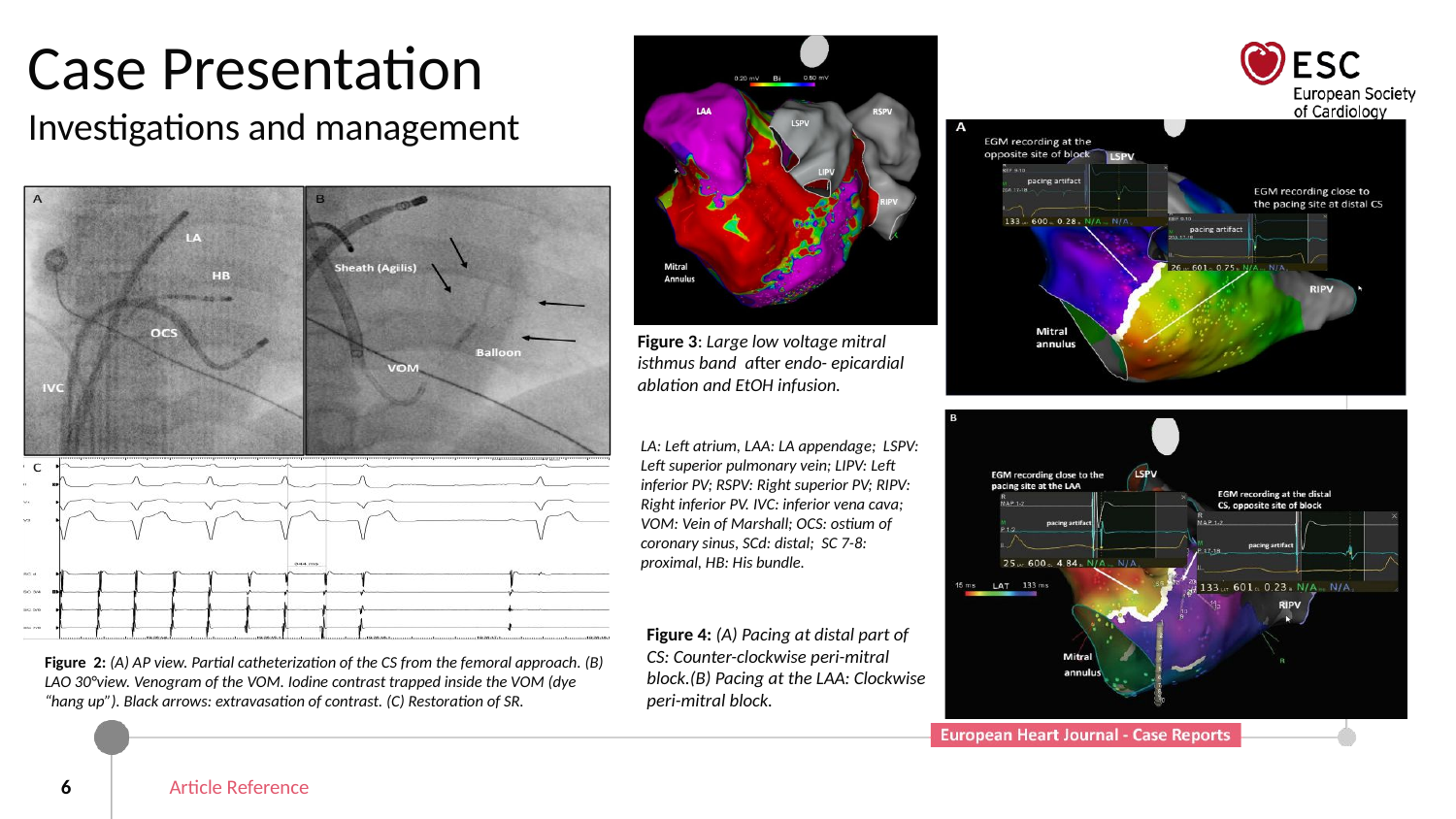

# Case Presentation Investigations and management
Figure 3: Large low voltage mitral isthmus band after endo- epicardial ablation and EtOH infusion.
LA: Left atrium, LAA: LA appendage; LSPV: Left superior pulmonary vein; LIPV: Left inferior PV; RSPV: Right superior PV; RIPV: Right inferior PV. IVC: inferior vena cava; VOM: Vein of Marshall; OCS: ostium of coronary sinus, SCd: distal; SC 7-8: proximal, HB: His bundle.
Figure 4: (A) Pacing at distal part of CS: Counter-clockwise peri-mitral block.(B) Pacing at the LAA: Clockwise peri-mitral block.
Figure 2: (A) AP view. Partial catheterization of the CS from the femoral approach. (B) LAO 30°view. Venogram of the VOM. Iodine contrast trapped inside the VOM (dye “hang up”). Black arrows: extravasation of contrast. (C) Restoration of SR.
6
Article Reference

## Slide 7
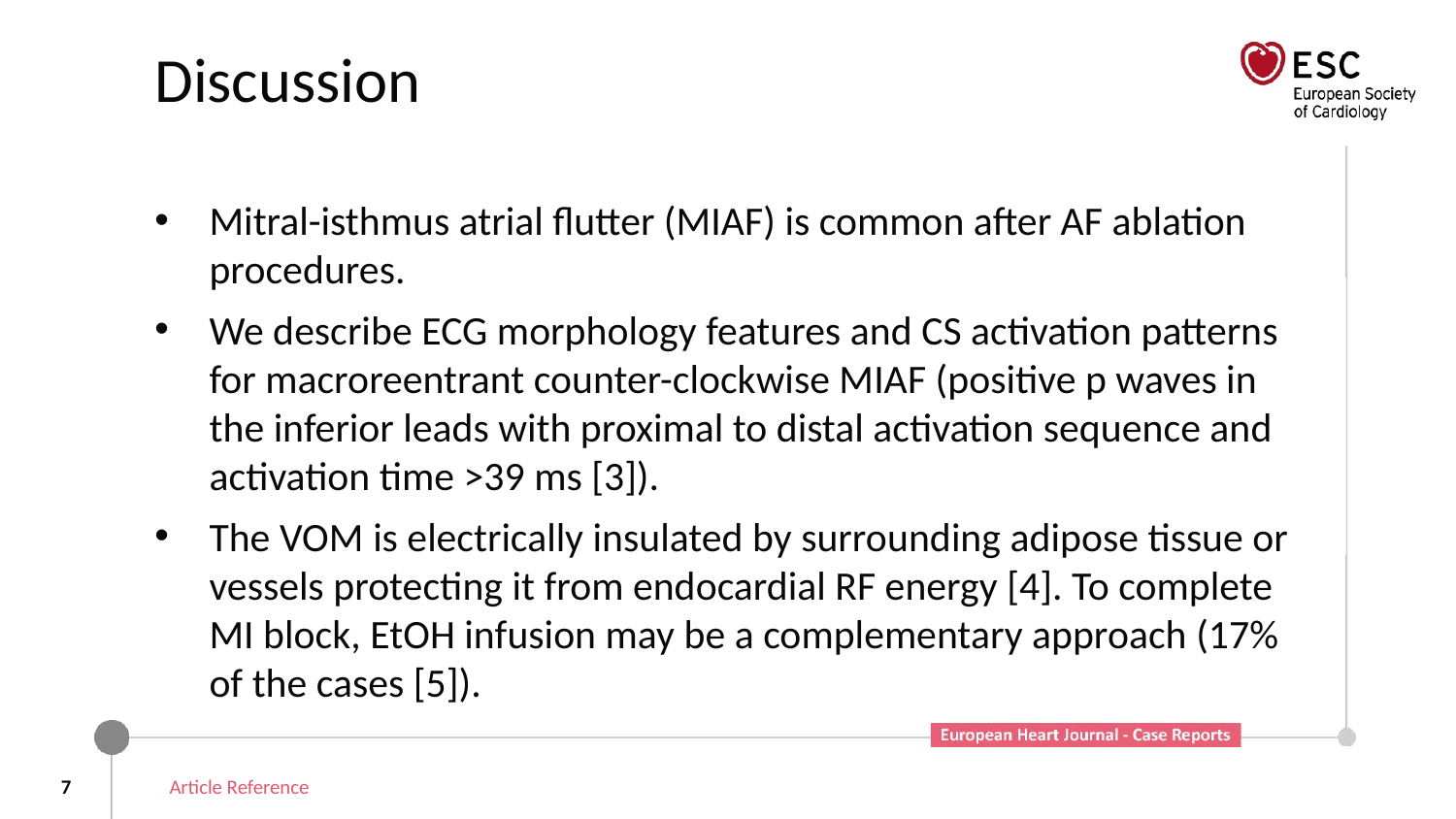

# Discussion
Mitral-isthmus atrial flutter (MIAF) is common after AF ablation procedures.
We describe ECG morphology features and CS activation patterns for macroreentrant counter-clockwise MIAF (positive p waves in the inferior leads with proximal to distal activation sequence and activation time >39 ms [3]).
The VOM is electrically insulated by surrounding adipose tissue or vessels protecting it from endocardial RF energy [4]. To complete MI block, EtOH infusion may be a complementary approach (17% of the cases [5]).
7
Article Reference

## Slide 8
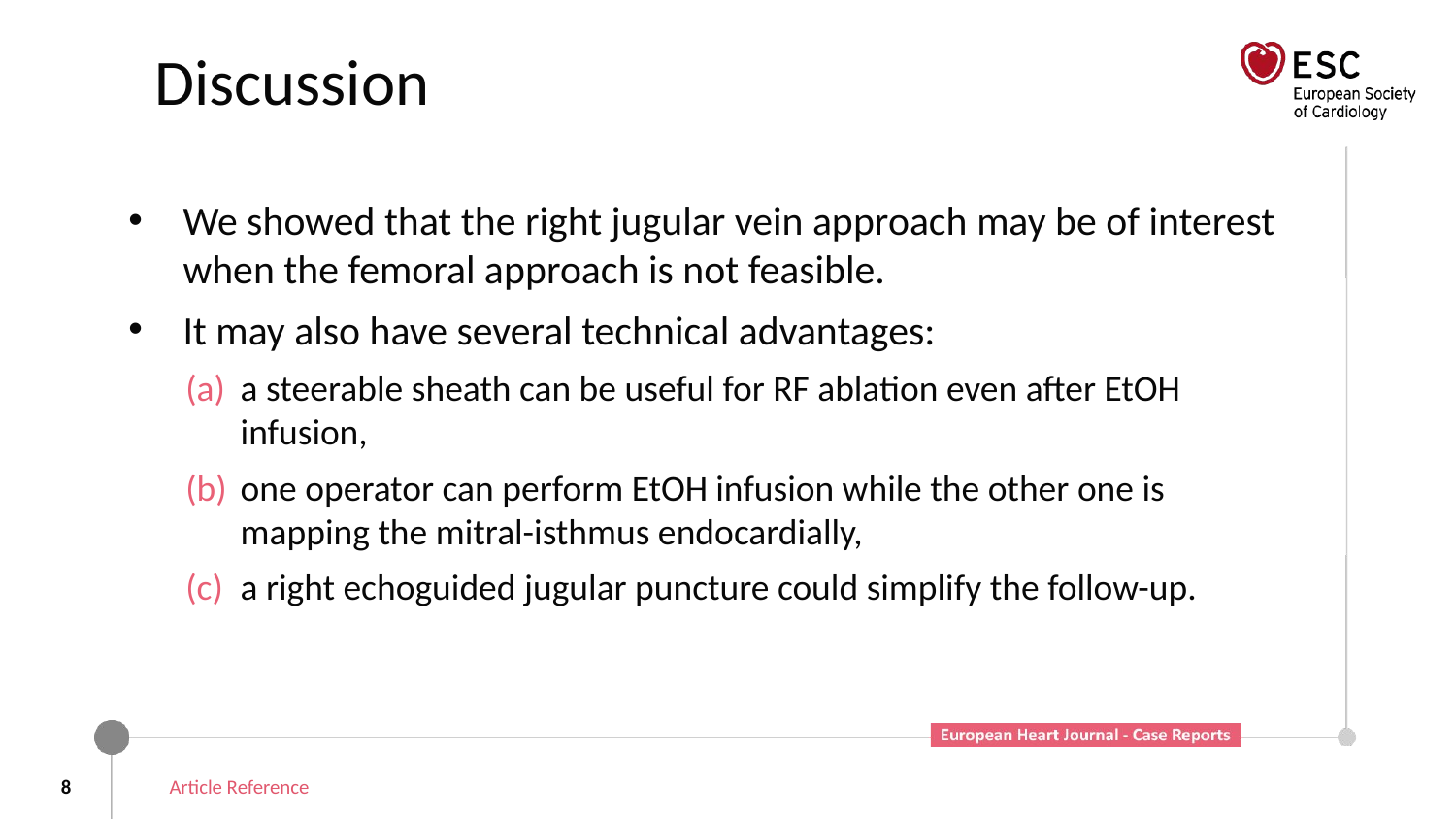

# Discussion
We showed that the right jugular vein approach may be of interest when the femoral approach is not feasible.
It may also have several technical advantages:
a steerable sheath can be useful for RF ablation even after EtOH infusion,
one operator can perform EtOH infusion while the other one is mapping the mitral-isthmus endocardially,
a right echoguided jugular puncture could simplify the follow-up.
8
Article Reference

## Slide 9
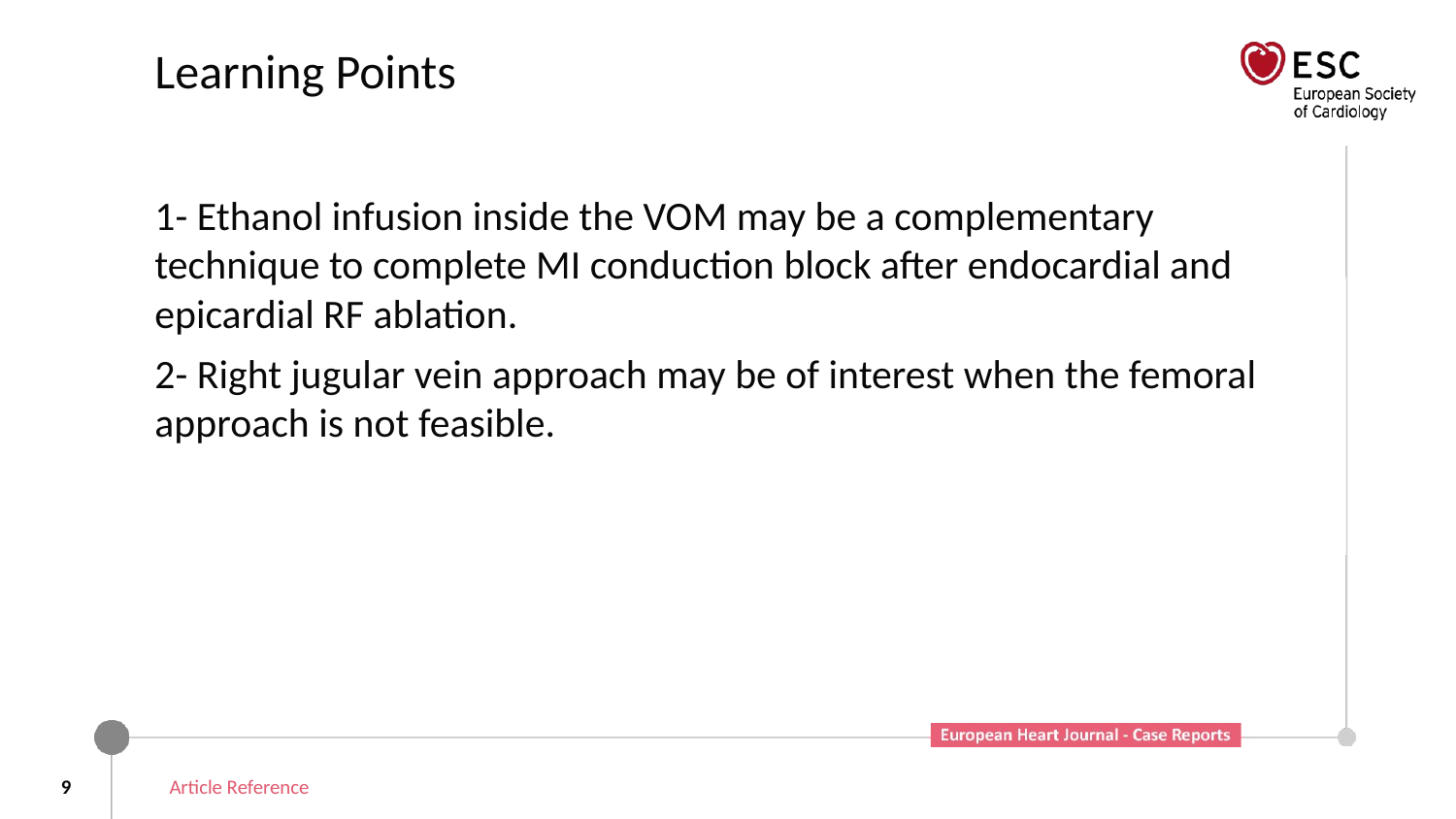

# Learning Points
1- Ethanol infusion inside the VOM may be a complementary technique to complete MI conduction block after endocardial and epicardial RF ablation.
2- Right jugular vein approach may be of interest when the femoral approach is not feasible.
9
Article Reference

## Slide 10
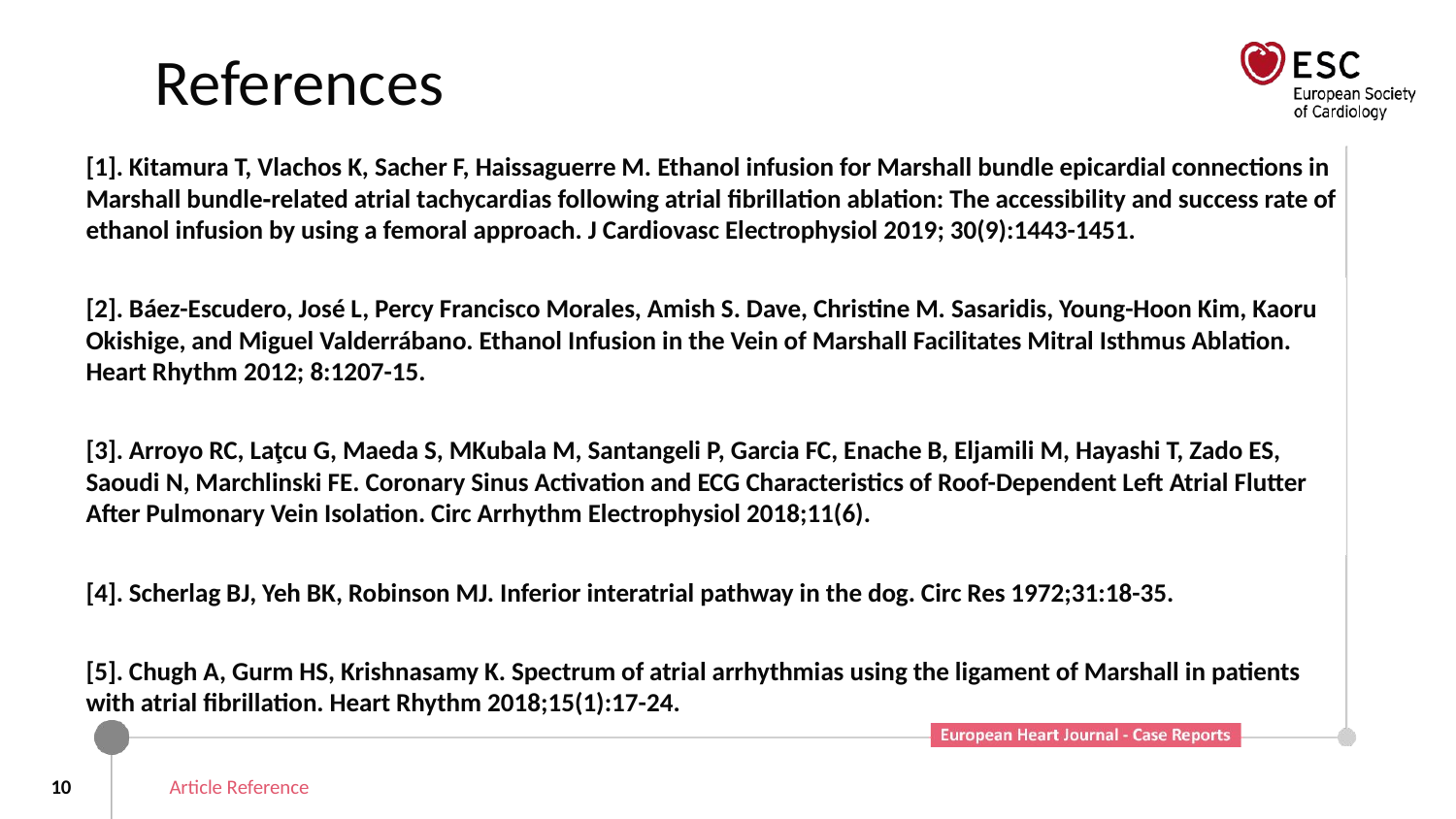

# References
[1]. Kitamura T, Vlachos K, Sacher F, Haissaguerre M. Ethanol infusion for Marshall bundle epicardial connections in Marshall bundle‐related atrial tachycardias following atrial fibrillation ablation: The accessibility and success rate of ethanol infusion by using a femoral approach. J Cardiovasc Electrophysiol 2019; 30(9):1443-1451.
[2]. Báez-Escudero, José L, Percy Francisco Morales, Amish S. Dave, Christine M. Sasaridis, Young-Hoon Kim, Kaoru Okishige, and Miguel Valderrábano. Ethanol Infusion in the Vein of Marshall Facilitates Mitral Isthmus Ablation. Heart Rhythm 2012; 8:1207-15.
[3]. Arroyo RC, Laţcu G, Maeda S, MKubala M, Santangeli P, Garcia FC, Enache B, Eljamili M, Hayashi T, Zado ES, Saoudi N, Marchlinski FE. Coronary Sinus Activation and ECG Characteristics of Roof-Dependent Left Atrial Flutter After Pulmonary Vein Isolation. Circ Arrhythm Electrophysiol 2018;11(6).
[4]. Scherlag BJ, Yeh BK, Robinson MJ. Inferior interatrial pathway in the dog. Circ Res 1972;31:18-35.
[5]. Chugh A, Gurm HS, Krishnasamy K. Spectrum of atrial arrhythmias using the ligament of Marshall in patients with atrial fibrillation. Heart Rhythm 2018;15(1):17-24.
10
Article Reference
